# Is Leadership a Resource? A Systematic Review of Its Role in Burnout and Engagement Among Nurses Within the JD-R Model

**DOI:** 10.1155/jonm/8853148

**Published:** 2025-11-27

**Authors:** Susana Montenegro Méndez, Ana Laguía González, Juan Antonio Moriano León

**Affiliations:** ^1^Department of Social and Organizational Psychology, Faculty of Psychology, Escuela Internacional de Doctorado, Universidad Nacional de Educación a Distancia (EIDUNED), Universidad Nacional de Educación a Distancia (UNED), C/Juan del Rosal, 10, Madrid 28040, Spain; ^2^Department of Social and Organizational Psychology, Faculty of Psychology, Universidad Nacional de Educación a Distancia (UNED), C/Juan del Rosal, 10, Madrid 28040, Spain

**Keywords:** burnout, Job Demands-Resources model, leadership, nurse, work engagement

## Abstract

**Introduction:**

Leadership significantly influences nurses' occupational well-being, particularly their levels of burnout and work engagement, according to the Job Demands-Resources (JD-R) model. This model posits that job demands increase burnout, whereas resources, such as leadership, promote work engagement.

**Objective:**

To analyze the influence of leadership styles on burnout and work engagement among nurses within the theoretical framework of the JD-R model.

**Methods:**

A systematic review was conducted following Preferred Reporting Items for Systematic Reviews and Meta-Analyses (PRISMA) guidelines between July and October 2024 in PubMed, CINAHL, WOS, SCOPUS, PROQuest, and PsycInfo. Quantitative observational studies focused on nurses and evaluating leadership, burnout, and work engagement within the JD-R model were included. A total of 38 relevant studies were identified and assessed for methodological quality using the STROBE tool.

**Results:**

Of the studies, 89.47 were cross-sectional and 10.53% were longitudinal. Transformational, authentic, and servant leadership styles demonstrated protective effects against burnout and promoted engagement. Transformational leadership emerged as the most frequently studied style, consistently associated with lower burnout levels and higher engagement. Conversely, negative styles such as abusive leadership were linked to increased burnout. Most studies used the Maslach Burnout Inventory (MBI) and the Utrecht Work Engagement Scale (UWES) to measure key variables.

**Conclusions:**

Effective leadership is a critical resource for improving nurses' well-being and performance. Investing in leadership development programs can reduce burnout and enhance engagement, benefiting both nurses and patients. Future studies should include longitudinal designs and explore innovative approaches, such as secure base leadership.

## 1. Introduction

Nurses are almost continuously exposed to psychosocial risk factors, including high quantitative demands, intense work rhythms due to the care of a large number of patients, and the performance of tasks unrelated to direct patient care. They also face emotional demands arising from caregiving activities and the constant confrontation with suffering, pain, and death. Additionally, they must balance paid work with domestic responsibilities, often working multiple jobs or engaging in shift work [1–4]. All these psychosocial risk factors can negatively impact their physical and mental health, as well as the quality of care they provide [1]. In this context, leadership plays a pivotal role, as it can serve as a workplace resource capable of mitigating the negative effects of these job demands and fostering a healthy work environment for nurses [5].

This need for protective organizational resources has become even more pressing in light of the worsening global nursing crisis. A 2019 report revealed that 61% of the nurses experience anxiety, depression, or burnout and 57% feel exhausted every day at work [6]. Nearly half have faced public aggression or violence simply for performing their duties. Alarmingly, a 10% rise in nurses' intention to leave the profession is associated with a 14% increase in patient mortality. The financial toll is immense, with declining care quality and rising medical errors in chronically understaffed and emotionally depleted teams. In this critical context, identifying leadership as a modifiable organizational resource becomes not only theoretically relevant but also a strategic priority to sustain nurse well-being and patient safety.

Further supporting this concern, a national study [7] reported that 88% of the nurses felt psychologically affected by workload pressure, 66.6% experienced anxiety, 60% suffered from insomnia, and 27.2% had symptoms of depression in recent months. Alarmingly, 6 out of 10 nurses stated they had considered leaving the profession. These increasing figures reflect the emotional toll of workload pressure and underline the need for organizational awareness and leadership action.

Complementing these findings, a large meta-analysis comprising 85 studies and 288,581 nurses across 32 countries [8], confirmed that nurse burnout is associated with a poorer safety climate and culture, lower safety standards, and a higher incidence of nosocomial infections, patient falls, medication errors, adverse events, and omitted or delayed care. It also found a consistent link between burnout and lower patient satisfaction, as well as reduced quality of care as perceived by nurses themselves.

In line with these findings, a systematic review has linked a supportive work environment with fewer burnout symptoms, less intention to leave work, and higher levels of job commitment. In addition, an adequate work environment was associated with lower stress and higher quality of care, social support, professional development, leadership, collaboration between nurses and physicians, nurse involvement in hospital affairs, staffing, and job satisfaction [9].

This landscape underscores the critical need to understand and address the drivers of nurse burnout and engagement. Effective leaders who can reduce workload pressure through proper team management, provide emotional support, promote autonomy, and facilitate access to essential organizational resources are becoming increasingly indispensable in healthcare institutions and organizations [10].

### 1.1. The Role of Leadership in Nurse Burnout and Engagement

Nurses are a critical component of the healthcare system, and their well-being directly impacts the quality of care provided to patients. Among the key factors influencing their performance and retention are burnout and work engagement, which have been shown to affect not only staff health but also patient satisfaction and safety. Burnout is associated with decreased job satisfaction, higher turnover, and reduced quality of care [11], while engagement has been linked to improved job performance, retention, and patient outcomes [12, 13].

Numerous theories aim to explain the key factors influencing nurses' professional lives, which, in turn, affect patient health outcomes. Some of these key factors include leadership, burnout, and work engagement [14–17]. When leaders demonstrate effective leadership techniques, nurses perceive a more supportive work environment, which can help them cope with work-related stress and burnout, ultimately contributing to the long-term retention of valuable human resources [1]. In this review, the term “leadership styles” refers specifically to behavioral approaches evaluated at the level of direct supervision, namely, immediate supervisory leadership (transformational, authentic, ethical, toxic, etc.) [18–21], understood as the behaviors of immediate supervisors or nurse managers who interact regularly with frontline staff and have a direct impact on their daily work experience [22].

The classical definition of burnout was formulated by Maslach and Jackson, who described it as a psychological syndrome characterized by emotional exhaustion, depersonalization, and reduced personal accomplishment, which can occur in, otherwise, healthy individuals [23]. This implies that when a professional is described as “burned out,” it reflects the fact that a work-related situation has overwhelmed them, depleting their ability to respond adaptively [24]. Although burnout was initially considered to be specific to professionals working in the care of people, later evidence has shown that this syndrome can develop among all types of professions and occupational groups [25].

Work engagement is a construct that emerged from positive organizational psychology, focusing on individuals' strengths. It is defined as “a positive mental state toward work, characterized by vigor, dedication, and absorption” [26]. Vigor refers to a high level of energy and willingness to invest effort in work, along with persistence in the face of challenges. Dedication involves being deeply committed to work activities, accompanied by feelings of enthusiasm, inspiration, pride, a sense of challenge, and meaning. Absorption is characterized by being fully focused and happily engrossed in one's work, to the extent that time passes quickly and there is a reluctance to leave the workplace [26].

### 1.2. The Job Demands-Resources (JD-R) Model as Theoretical Framework

Initially, work engagement was considered the direct opposite of burnout [27]. Subsequently, the JD-R theory [28, 29] provided a more integrated explanation, proposing that burnout and work engagement are distinct, yet interconnected processes influenced by job demands and resources. The JD-R theory posits that employee well-being is shaped by the interaction between job demands and resources through two interrelated psychological processes: one leading to burnout via the strain caused by job demands, and the other fostering engagement through the availability of job resources [29].

The JD-R theory explains how job demands and resources influence work performance through employee well-being, encompassing both burnout and engagement. It also highlights how employees employ proactive and reactive work behaviors to shape job demands and resources. This theory is flexible, capable of integrating a wide range of job characteristics, and synthesizes concepts from other theories and models related to stress, motivation, and occupational well-being. It posits that burnout and work engagement can result from diverse job characteristics [30].

According to the JD-R theory, workplace resources exist at four levels: organizational (e.g., career opportunities); work organization (e.g., participation in decision-making); interpersonal and social relationships (e.g., supervisor support or leadership); and task-level (e.g., skill variety) [28]. Job demands, on the other hand, encompass the social, psychological, physical, and organizational aspects of a job that require sustained physical or mental effort, leading to psychological or physiological effects [28]. Examples of job demands for nurses include physical demands, emotionally challenging interactions with clients, and harmful work environments [31].

The multilevel approach of the JD-R theory acknowledges that nurses are embedded in teams, which, in turn, are part of larger organizations. At the highest organizational level, senior management plays a critical role in shaping the strategic function of human resources and the organizational climate [30]. Through several human resource practices, organizations can select and develop leaders who influence the job demands and resources of their teams, thereby indirectly affecting employee well-being and performance [32].

Given the centrality of burnout and engagement in understanding how working conditions affect nurse well-being and care quality, this systematic review focuses on these two constructs as defined within the JD-R model. Specifically, it examines the associations between different leadership styles and levels of burnout and engagement among nurses, with the aim of identifying consistent patterns across the existing literature.

Although numerous individual studies have examined the relationship between leadership styles and nurses' well-being, the evidence remains fragmented due to variability in leadership models (e.g., transformational, transactional, authentic, servant, and toxic), measurement tools, and outcome indicators. This heterogeneity hinders the development of evidence-based recommendations for healthcare management. Therefore, the present review aims to synthesize available findings using the JD-R model as a guiding framework. By classifying leadership styles according to their behavioral profiles and assessing the methodological quality of the included studies, the review seeks to identify which leadership approaches are most effective in reducing burnout and promoting engagement in nursing contexts. This integration will facilitate the translation of theoretical insights into actionable strategies for practice and policy.

## 2. Materials and Methods

### 2.1. Selection Criteria

A systematic review of the scientific literature was conducted between July and October 2024, following the Preferred Reporting Items for Systematic Reviews and Meta-Analyses (PRISMA) guidelines [33]. Bibliographic searches were performed in the following databases: PubMed, Cumulative Index of Nursing and Allied Literature (CINAHL), Web of Science (WOS), SCOPUS, PROQuest, and PsycInfo.

The search aimed to answer the following question: Within the theoretical framework of the JD-R theory, does leadership style influence burnout and work engagement among nurses? This question was structured according to the PICO framework [34]:

- Population (P): nurses
- Intervention (I): leadership style
- Comparison (C): not applicable (no comparative group included),
- Outcome (O): burnout and work engagement.

The study selection process was conducted by the lead author, who thoroughly reviewed the titles and abstracts of the identified publications. In cases of uncertainty regarding the inclusion or exclusion of a study, these were discussed and resolved in consensus with the coauthors. This ensured a uniform and rigorous selection process, guaranteeing the validity and reliability of the data collected for subsequent analysis in this systematic review.

### 2.2. Search Strategy

Medical Subject Headings (MeSH) terms used included “Burnout, Professional,” “Work Engagement,” “Nurses,” and “Leadership.” Equivalent MeSH terms for the JD-R model were not identified. It is important to note that this specific model, originating from organizational psychology, does not have an exact classification within MeSH, as it pertains more to the field of occupational and organizational psychology than to pure medical sciences.

As the objective of the scientific literature review was to identify studies analyzing the association of leadership on burnout and work engagement among nurses within the theoretical framework of the JD-R theory, the following keyword equation was used for searches across all databases: (job demand and resources model OR job demand and resources theory OR job-demand resources theory OR JD-R model) AND (burnout [Mesh] OR engagement [Mesh] OR work engagement [Mesh]) AND (nurse [Mesh] OR nursing [Mesh]) AND leadership [Mesh].

In addition to the articles identified through the systematic search in the selected databases, additional publications were included by reviewing the reference lists of the selected studies. This approach broadened the scope of relevant literature, ensuring that no key studies related to the topic were overlooked.

### 2.3. Inclusion Criteria

Full-text scientific articles published in peer-reviewed journals between January 2006 and June 2024 (quantitative, qualitative, or mixed-methods), written in English or Spanish. The studies had to include nurses as participants and analyze the association of leadership on burnout and/or work engagement within the theoretical framework of the JD-R theory.

### 2.4. Exclusion Criteria

Articles not published in peer-reviewed journals (e.g., conference abstracts, editorials, letters to the editor, opinion pieces, technical reports, or unpublished theses), studies not available in full text, publications in languages other than English or Spanish, studies that did not include nurses as the primary group of participants, studies that did not evaluate the association of leadership on burnout and/or work engagement, purely theoretical studies without empirical components (e.g., narrative reviews or conceptual essays), and research that did not employ quantitative, qualitative, or mixed methods.

### 2.5. Data Extraction

Following the quality criteria outlined in the PRISMA statement, data extraction was carried out in several phases. In the first phase, the records identified during the search were sequentially imported into the reference management software Mendeley (Version 2.122.0), where duplicates across different databases were identified and removed. In the second phase, the titles and abstracts of the screened articles were reviewed (or full texts when necessary), and those not meeting the inclusion criteria were excluded. Finally, a critical reading of the selected articles was performed to extract the following information: author, year of publication, country of origin, study type, sample characteristics (number of participating nurses, study setting or nursing specialty analyzed, gender, and age), hypotheses or objectives, analyzed variables, instruments used for variable analysis, results, and conclusions.

### 2.6. Presentation of the Results

**Figure 1:**
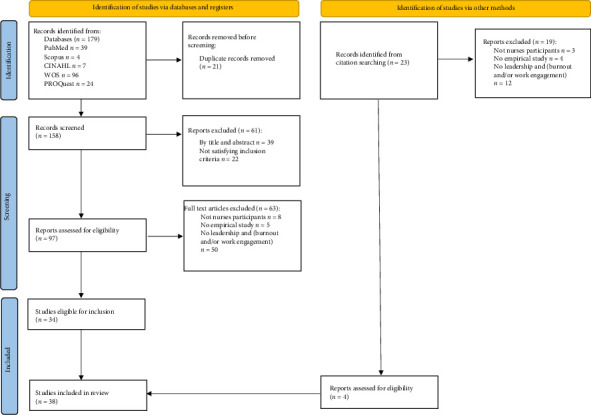
PRISMA flowchart.

Following the search strategy and selection criteria (Figure 1), 38 articles [31, 35–71] were included in this systematic review, all published in English. Of these, 18 articles utilized conceptual integrations as their framework [44, 47, 48, 54, 56, 61, 66, 67, 69] or theoretical models other than the JD-R model [38, 49, 50, 55, 57, 64, 66–68, 70, 71]; however, these studies specifically analyzed the variables of interest for this review: leadership, burnout, and engagement. The inclusion of these studies was justified by their relevance in addressing the main objective of the analysis and their contribution to a comprehensive understanding of the relationships between these variables.

### 2.7. Quality Evaluation

Since all the selected studies were quantitative observational designs, their methodological quality was assessed using the Strengthening the Reporting of Observational Studies in Epidemiology (STROBE) checklist. Studies scoring ≥ 14 out of 22 were considered to have good quality [72].

The levels of evidence were also evaluated following the Joanna Briggs Institute guidelines, with classifications as follows: 1 for evidence from experimental designs, 2 for quasiexperimental designs, 3 for analytical observational studies, 4 for descriptive observational studies, and 5 for consensus documents and expert opinions [73].

Although a meta-analytic synthesis was initially considered, it was not feasible due to the substantial heterogeneity across studies in terms of leadership constructs, measurement instruments, and reported outcomes. Specifically, the included studies used diverse leadership frameworks (e.g., transformational, authentic, and servant), varying definitions and measures of burnout and engagement (e.g., Maslach Burnout Inventory [MBI], Utrecht Work Engagement Scale [UWES], and single-item indicators), and reported results in different statistical formats, thus hindering the possibility of computing pooled effect sizes. Therefore, a narrative synthesis was deemed more suitable to capture the complexity and contextual nuances of the findings.

## 3. Results

Table 1 summarizes the information obtained from these studies.

Of the 38 studies, 89.47% (*n* = 34) [31, 36, 37, 39, 40, 42–56, 58–71] were cross-sectional quantitative observational studies, while 10.53% (*n* = 4) [35, 38, 41, 57] were prospective longitudinal quantitative observational studies.

Regarding the temporal distribution, 57.90% (*n* = 22) of the studies [31, 35–55] were conducted between 2021 and 2024, while the remaining 42.10% (*n* = 16) [56–71] were distributed over the preceding 14 years (2009–2020).

The geographical distribution of the studies shows a higher concentration in Canada [47, 50, 63, 64, 66, 67, 71] (*n* = 7), followed by the United States [39, 52, 56] and China [35, 38, 48] (*n* = 3). Other countries contributing two studies each include Pakistan [55, 58], Belgium [36, 69], Italy [59, 70], Germany [42, 53], Turkey [37, 49], and Finland [45, 62]. Additionally, the review includes single studies from various countries, such as Ireland [31], Portugal [68], Romania [57], Cyprus [43], Australia [48], Brazil [54], Israel [51], Taiwan [60], India [46], Malta [41], the United Arab [44], Iran [65], Myanmar [40], and Nigeria [61]. The analysis of the number of participants in the studies reveals the following results: a total of *n* = 47,569 participants, an average of 1219 participants per study, with the largest study including 16,191 participants and the smallest study including 89 participants.

The included studies cover a wide range of nursing profiles. The included studies cover a wide range of nursing profiles. A total of 60.52% of the studies [31, 35–38, 40, 41, 43, 46, 48, 51, 54, 55, 57–61, 65, 66, 69–71] (*n* = 23) focus on hospital nurses, while others [39, 44, 45, 49, 50, 52, 53, 56, 62, 63, 68] (28.94%) (*n* = 11) examine nurses in other healthcare institutions or facilities. Additionally, studies include newly graduated nurses [64, 67] (*n* = 2), nurse managers [42] (*n* = 1), and nursing faculty members in academic institutions [47] (*n* = 1). Additionally, studies include newly graduated nurses [64, 67] (*n* = 2) and nursing faculty members in academic institutions [47] (*n* = 1). The average age of nurses across the studies is 37.06 years, and the average percentage of female nurses is approximately 84.64%.

Of the 38 studies analyzed, 18.42% [31, 36, 39, 41, 51, 57, 71] (*n* = 7) examined all three variables of this systematic review (leadership, burnout, and engagement or work engagement). Additionally, 44.74% [38, 45, 47–50, 52, 54–56, 59, 60, 63, 64, 68–70] (*n* = 17) focused solely on leadership and burnout, while 36.84% [35, 37, 40, 42–44, 46, 53, 58, 61, 62, 65–67] (*n* = 14) on leadership and work engagement.

Transformational leadership is one of the most frequently studied styles, analyzed in 31.58% (*n* = 12) of the studies [35, 40, 41, 44, 47, 53, 58, 61–63, 65, 68]. This is followed by supportive leadership in 15.79% (*n* = 6) of the studies [42, 43, 46, 48, 54, 69], authentic leadership in 10.53% (*n* = 4) [37, 64, 66, 67], servant leadership in 7.89% (*n* = 3) [38, 52, 55], effective leadership in 7.89% (*n* = 3) [50, 59, 60], and empowering leadership in 5.26% (*n* = 2) [42, 71]. Other leadership styles analyzed include ethical leadership [31] and committed leadership [36].

Of the studies analyzing the burnout variable, 75% (*n* = 18) used the MBI [31, 38, 41, 47–51, 54–56, 59, 63, 64, 67, 69–71]. The Burnout Assessment Tool (BAT) was employed in 8.3% (*n* = 2) of the studies [36, 45]. The BAT, used in some more recent studies, evaluates burnout with a stronger focus on job demands and resources. Other instruments used to analyze burnout included the All Employee Survey [39], Westbrook et al.'s scale [52], and Chen's adapted scale [60].

Of the studies analyzing work engagement or engagement, 76.19% (*n* = 16) used the UWES to measure this variable [31, 35–37, 40–42, 46, 51, 53, 58, 61, 62, 65, 66, 68]. Among these, four studies [36, 41, 42, 46] used the ultrashort version (UWES-3), seven studies [31, 37, 51, 53, 61, 62, 66] used the short version (UWES-9), and five studies [35, 40, 58, 65, 68] used the original version (UWES-17). Other instruments employed to analyze work engagement included the All Employee Survey [39], a survey based on the NHS England Staff Survey [43], and the Hasan et al.'s scale [44].

Other relevant variables analyzed in the selected studies included psychological safety [38, 55], work environment [42, 44, 53, 54, 70], turnover intention [31, 39, 52, 57, 60, 69], and job satisfaction [35, 52, 54, 56, 57, 60].

All studies analyzing positive or effective leadership styles [31, 35–44, 46–48, 51–71] (e.g., transformational, ethical, supportive, authentic, servant, committed, and proactive) consistently report their protective effect on burnout and their positive influence in fostering engagement among nurses. Conversely, all studies examining negative leadership styles [45, 49, 50] (e.g., abusive, destructive, and toxic) associate these styles with higher levels of burnout among nurses; none explore their association on work engagement. Transformational leadership stands out as one of the most frequently studied styles, consistently linked to reduced burnout levels and increased engagement.

All reviewed studies [31, 35–71] achieved high scores on the STROBE scale, ranging from 18/22 to 21.5/22, indicating solid methodological quality overall. The level of evidence was categorized as “4b” in 89.47% of the studies [31, 36, 37, 39, 40, 42–56, 58–71] and “3c” in 10.52% of the studies [35, 38, 41, 57], reflecting moderate to good quality of evidence. The findings of this analysis are shown in Table 2.

## 4. Discussion

The results of this systematic review reveal a clear influence of leadership on burnout and work engagement among nurses, consistent with the JD-R theory. Positive leadership styles (e.g., transformational, supportive, authentic, and servant) demonstrate a protective effect against burnout and promote nurses' work engagement. In contrast, studies analyzing negative leadership styles (e.g., abusive, destructive, and toxic) associate these styles with higher levels of burnout among nurses. This finding underscores the critical need to promptly identify and address harmful leadership practices in nursing environments.

Transformational leadership emerges as one of the most extensively studied styles, consistently associated with reduced levels of burnout and increased engagement. This leadership style [74], characterized by inspiring and motivating the team, appears particularly effective in fostering a positive work environment and promoting professional development. Authentic and servant leadership are also identified as influential in enhancing nurses' well-being, as they foster trust and psychological safety, which act as mediators in reducing burnout.

It is also noteworthy, especially within the JD-R framework, that none of the studies examining negative leadership styles (e.g., abusive or toxic leadership) investigated their impact on work engagement, despite the theory's established premise that such detrimental behaviors function as job demands or hindrance stressors that deplete resources and undermine engagement.

Although most of the studies included evaluated the existence of an association between leadership and levels of burnout or engagement, several studies go a step further and describe how this effect occurs. In line with the JD-R model [29], positive leadership not only acts as a direct organizational resource but also enhances other resources that mitigate exhaustion and promote engagement. Leadership acts not only as a direct predictor but also as a generator of resources (personal and organizational) that cushion the negative effects of work demands and enhance professional commitment [75].

For example, Spence Laschinger et al. find that authentic leadership increases nurses' self-efficacy and sense of competence, which in turn reduces their levels of burnout [67], Zaghini et al. demonstrate that satisfaction with management increases psychological safety, fostering a supportive environment where demands have less impact on burnout [59], and Ding and Cao describe how transformational leadership improves engagement by strengthening team cohesion and communication, which reduces the perception of overload [35].

Nevertheless, research on emerging leadership approaches, such as secure base leadership, remains limited [76]. This leadership style has been associated with lower levels of burnout and higher levels of engagement in other sectors [76, 77], and its study is considered particularly relevant for future nursing research due to its positive influence on psychological safety climates [77, 78], a variable identified in several studies in this review [38, 55].

The pandemic has marked a turning point in healthcare systems and organizations, forcing nurses to face new challenges in a very short period of time, adapt their roles and competencies, and update existing strategies and protocols [5]. This pivotal moment is reflected in the significant increase in the number of studies on this topic in recent years, with 57.90% of the analyzed studies concentrated between 2021 and 2024. The predominance of cross-sectional quantitative observational studies (89.47%) highlights a clear methodological trend. However, this prevalence suggests the need for more longitudinal research to establish stronger causal relationships, as observed in the longitudinal studies reviewed [35, 38, 41, 57].

The broad diversity of countries represented in this systematic review highlights the international interest in the relationship between leadership, burnout, and work engagement in the nursing field, evidencing the global nature of this issue. Furthermore, the diversity of nursing profiles and work settings analyzed allows for the exploration of how the work context influences the association of leadership on burnout and work engagement across different areas of the nursing profession.

While the MBI and UWES are widely used, the variety of versions (e.g., MBI-HSS vs. MBI-GS; UWES-3, UWES-9, and UWES-17) and the different subscales or dimensions selected across studies have precluded direct comparability of results. However, regarding burnout, an increasing number of studies are beginning to adopt the BAT, as evidenced in recent studies by Kohnen et al. [36] and Palvimo et al. [45]. This shift reflects a change in the approach to measuring burnout, aligning more closely with the JD-R theory.

In spite of its contribution, this study presents several limitations in its design and approach that should be considered when interpreting the findings. First, most of the included studies employed a cross-sectional observational design, which hinders the establishment of causal relationships between leadership and the analyzed variables (burnout and engagement). Although some longitudinal studies provide valuable data, their representation is limited compared to cross-sectional designs. Second, the review was restricted to articles published in English and Spanish, potentially excluding relevant research in other languages. Additionally, despite using an exhaustive search strategy across scientific databases, there is a possibility of publication bias, as unpublished studies or those with nonsignificant results may not be represented. Third, while the review encompasses a wide variety of contexts and countries, cultural and organizational differences may affect the generalizability of the results, particularly regarding the relationship between leadership styles and psychosocial variables in nursing settings. Finally, the extreme heterogeneity, both in leadership styles and in the instruments and dimensions of burnout and engagement, not only prevented a quantitative meta-analysis but also highlights the lack of consensus in the field, making it difficult to accumulate solid evidence and compare studies. It is essential to move toward unifying definitions and measures in order to obtain more robust conclusions.

These limitations highlight the need for future longitudinal and multicenter studies, as well as the inclusion of greater cultural diversity and methodological approaches, to strengthen the available evidence on this topic.

As highlighted in the conclusions of studies by Olmos et al. [79, 80], the findings of this systematic review suggest that developing leadership competencies in nursing education and practice could strengthen the sociopolitical role of the profession and address current challenges in healthcare.

In line with other research [81, 82], this review reinforces the need for healthcare institutions to prioritize supportive and effective leadership styles among nursing supervisors, alongside measures to promote work–life balance, in order to enhance staff retention and reduce emotional exhaustion.

Given the global shortage of nursing professionals, designing and implementing strategies that safeguard nurses' well-being and strengthen their commitment to the profession is essential. Investing in positive and efficient supervisory leadership represents one of the most impactful and feasible measures in this direction.

## 5. Conclusions

This systematic review provides robust evidence on the importance of leadership in managing burnout and promoting work engagement in nursing, supporting the applicability of the JD-R theory in this context. The findings emphasize the need to prioritize the development of effective leadership as a strategy to enhance nurses' well-being and performance.

Scientific evidence confirms that effective and positive leadership styles are a key and indispensable resource in the nursing profession. They reduce burnout and foster engagement, which are crucial for improving the quality of the work environment and, consequently, patient care. The implementation of positive and effective leadership styles that value both job resources and the psychological health of nurses can play a fundamental role in retaining and satisfying nursing staff.

Based on the analysis of the reviewed studies, several recommendations can be made for healthcare institutions and facilities. Promoting and implementing leadership development programs tailored specifically for nurses could be an effective strategy to reduce burnout and increase work engagement, benefiting both nurses and the quality of care provided. Leaders who focus on strengthening job resources and providing effective support while fostering a climate of psychological safety can significantly enhance nurses' engagement. This not only improves their job satisfaction but also increases their willingness to remain in their roles and to actively contribute to creating a better work environment.

This systematic review underscores the critical role of leadership in addressing the psychosocial challenges faced by nurses, providing a foundation for the design of targeted interventions and institutional policies aimed at promoting healthier and more sustainable healthcare work environments.

To move beyond descriptive associations, future research should aim to (1) standardize the measurement of leadership styles and well-being outcomes to enable meta-analytic synthesis; (2) adopt longitudinal and intervention-based designs to test causal effects of leadership development programs; (3) conduct multilevel analyses that differentiate between immediate supervisory behaviors and broader organizational leadership; (4) explore diverse care contexts—including primary, long-term, and low-resource settings—to identify contextual moderators; and (5) examine potential mediating mechanisms, such as psychological safety or professional self-efficacy, through mixed-methods approaches. Advancing along these lines will enable the translation of theoretical insights into evidence-based strategies to improve both nurse well-being and quality of care.

## Figures and Tables

**Table 1:** Characteristics of the studies included in the systematic review.

Authorship Year Country	Study design	*N* Area of work Female % Average age	Study variables	Instrument(s)	Results	Conclusions	Quality level of evidence
Ding and Cao [35] 2024 China	2-waves longitudinal prospective quantitative study	• 292 • Hospital nurses • 97.6% • 29.18	• Work engagement • Transformational leadership • Hindrance job stressor • Challenge job stressor • Overall satisfaction • Satisfaction with service	• Utrecht work engagement scale • Leadership scale developed by Podsakof et al. (1996) • Frequency of overtime work • Chan et al. (2010) client participation scale adapted to the healthcare context • 2 items overall satisfaction scale • SERVQUAL scale from Parasuraman et al. (1988)	A substitutive interaction was found between transformational leadership and job demands (perceived as challenging stressors) in the relationship with nurses' work engagement	Results reveal a substitutive interaction between transformational leadership and challenge stressor, manifested in workload demands, for nurses' engagement, such that work load demands substitute for the effect of transformational leadership on nurses' engagement	STROBE: 21.5/22 JBI: 3c

Kohnen et al. [36] 2024 Belgium	Cross-sectional quantitative study	• 1117 • Hospital nurses • 82% • 40	• Engaging leadership • Job demands and resources • Intrinsic motivation • Burnout • Work engagement	• Engaging leadership scale • Energy compass Psychometric instrument • Work extrinsic and intrinsic motivation scale • Burnout assessment tool • Utrecht work engagement scale	Engaging leadership was associated with lower levels of burnout in nurses. Engaging leadership was also related to greater work commitment	Engaging leaders foster a favorable work environment for nursing staff which is not only beneficial for their work motivation but also for their work-related well-being	STROBE: 20/22 JBI: 4b

Uluturk et al. [37] 2024 Turkey	Cross-sectional quantitative study	• 597 • Hospital nurses • 85.9% • 30.41	• Authentic leadership • Psychological empowerment • Motivating language • Work engagement	• Authentic leadership inventory by Neider y Schriesheim (2011) • Spreitzer's psychological empowerment scale (1995) • Motivating language scale by Mayfield y Mayfield (2007) • Utrecht work engagement scale	Authentic leadership positively affects work engagement directly and indirectly through motivating language and psychological empowerment	Authentic leadership influences employee work engagement	STROBE: 19/22 JBI: 4b

Ahmed et al. [38] 2023 China	3-waves longitudinal prospective quantitative study	• 1204 • Hospital nurses • 81.3% • 38.2	• Servant leadership • Psychological safety • Trust in the leader • Burnout	• Liden et al. (2015) servant leadership scale • Detert and Burris (2007) adapted psychological safety scale • MacKenzie et al. (2001) trust in the leader scale • Maslach burnout inventory-general survey	Servant leadership has a direct negative association with nurses' burnout. Trust in the leader acts as a moderator between servant leadership and employee burnout	Servant leadership decreases burnout, especially when there are high levels of trust in the leader	STROBE: 21/22 JBI: 3c

Apaydin et al. [39] 2023 USA	Cross-sectional quantitative study	• 16,191 • Primary care nurses • 74.5% • 49.4	• Burnout • Protective factors: turnover intention, employee engagement, perceptions of workload, leadership and workgroup • Healthcare system-level predictors of burnout and turnover intention • Healthcare system-level COVID-19 burden, virtual care use, and prior year average HCW burnout and turnover intent	• The all employee survey is a yearly survey of primary care employee	Highly engaged employees were less burned out and had lower turnover intent. Individual perceptions of reasonable workload, high quality leadership, and good workgroups were linked with lower rates of burnout and turnover intent. Only workload had a stronger relationship with burnout than engagement, suggesting that engagement may encompass aspects of the working environment that are more than just the sum of workload, leadership, and workgroups	Employee engagement was associated with a lower likelihood of primary care HCW burnout and turnover intent during the pandemic, suggesting it may have a protective effect during stressful times. COVID-19 burden and virtual care use were not related to either outcome	STROBE: 19.5/22 JBI: 4b

Aung Po et al. [40] 2023 Myanmar	Cross-sectional predictive quantitative study	• 512 • Hospital nurses • 99.37% • 30.41	• Role performance • Job demands: work pressure, cognitive demands, emotional demands role and hassles • Job resources: autonomy social support, feedback, possibilities for growth and coaching • Personal resources • Work engagement • Job crafting resources • Transformational leadership	• Job performance scale by Williams and Anderson (1991) • Job demands scale by Bakker and Demerouti (2014) • Job resources scale by Bakker and Demerouti (2014) • Personal resources scale by Bakker and Demerouti (2014) • Work engagement scale by Bakker and Demerouti (2014) • Job Crafting scale by Tims et al. (2012) • Global transformational leadership scale by Carless et al. (2000)	Job resources and transformational leadership could not predict job performance in this study. Work engagement was an important predictor of nurses' job performance	This study identifies personal resources, job demands, work engagement, and job crafting as significant predictors of nurses' job performance in Myanmar, offering valuable insights for nursing administrators to enhance working environments and improve performance outcomes.	STROBE: 19.5/22 JBI: 4b

Buttigieg et al. [41] 2023 Malt	2-waves longitudinal prospective quantitative study	• 623 • Hospital nurses • 53.3% • 36.6	• Appraisal of organizational change as a demand (AOCD) • Transformational leadership • Work engagement • Burnout	• Appraisal of organizational change as a demand scale (AOCDP) • Rafferty and Griffin's transformational leadership scale (2004) • Utrecht work engagement scale • Maslach burnout inventory-human Service	The results show that the more employees value organizational change as a job requirement at time 1, the more burnout and less engagement they show at time 2, but not vice versa, and that transformational leadership maintained engagement but did not dampen burnout	Organizations facing major change should be aware of the real danger that their employees can get burnt out and become less engaged. Employees' appraisal of the change as job demands seems to play an important role herein	STROBE: 21/22 JBI: 3c

Forster and Koob [42] 2023 Germany	Cross-sectional quantitative study	• 438 • Nurse managers • 70.3% • 44.1	• Work engagement • Job resources: professional resources, supervisor support, effective relationships with nursing staff, collegial relationships with medical staff, empowering leadership, interpersonal relations between colleagues and transparent information and communication • Job demands: lack of formal rewards, work overload, work–life interferences, emotional demands, role conflicts	• Utrecht work engagement scale • Job resources instruments: nurse manager practice environment scale, Copenhagen psychosocial questionnaire and Haynes et al.'s autonomy and control scale • Job demands instruments: effort-reward imbalance questionnaire and Copenhagen psychosocial questionnaire	Empowering leadership was significantly and positively associated with work engagement	Empowering leadership should be promoted in the work environment of nurse managers. Nurse managers should be provided engaging financial and nonfinancial rewards	STROBE: 20/22 JBI: 4b

Giallouros et al. [43] 2023 Cyprus	Cross-sectional quantitative study	• 348 • Hospitals and health centers nurses • 70% • 38.5	• Job resources: training and development opportunities, line management resources, shared organizational vision • Leadership factors: leadership role encouragement, senior management's effective communication and involvement • Employee engagement: including dedication, vigor, absorption	• Survey based on the NHS England staff survey (2018) to assess employee engagement, focusing on dimensions like dedication, vigor, absorption, advocacy, and influence in decision-making	Leadership factors, such as leadership role support and employee orientation, foster greater engagement by enhancing job resources. Shared organizational vision stood out as a key resource to increase commitment	Managers can improve employee engagement in the public healthcare sector by focusing on strengthening workforce resources through effective leadership	STROBE: 19.5/22 JBI: 4b

Hasan et al. [44] 2023 Arab emirates	Cross-sectional quantitative study	• 353 • Healthcare clinics • 66.5% • 40.4	• Transformational leadership • Work engagement • Task performance, interpersonal facilitation and job dedication • Work environment	• Transformational leadership scale adapted from Ayadi and Khalil (2019) • Work engagement scale developed by Al-Buthi (2018) • Aleid's job performance scale (2018) • Work environment scale developed by Riyanto et al. (2021)	The relationship between transformational leadership and job performance among nurses in healthcare clinics was strongly influenced by work engagement. Transformational leadership had a positive role in work engagement	Transformational leadership and work engagement positively impact nurses' job performance, with the work environment acting as a moderator. Enhancing leadership strategies and fostering engagement can significantly improve performance outcomes in healthcare settings	STROBE: 19.5/22 JBI: 4b

Palvimo et al. [45] 2023 Finland	Cross-sectional quantitative study	• 2115 • Health and social care nurses • 93% • 46	• Burnout • Destructive leadership • Job demands and resources	• Burnout assessment tool • Destructive leadership scale • Adaptation of items from the European working conditions surveys	Destructive leadership is associated with burnout among registered nurses	To improve leadership quality within the nursing profession, it is essential to recognize and acknowledge destructive leadership styles in healthcare organizations	STROBE: 19.5/22 JBI: 4b

Uddin [46] 2023 India	Cross-sectional quantitative study	• 259 • Hospital nurses • 73% • 44.15	• Perceived supervisory support • Perceived coworkers support • Work engagement • Affective commitment	• Supervisor support scale adapted from Karasek et al. (1982) • Peer support scale adapted from Karasek et al. (1982) • Utrecht work engagement scale • Affective commitment scale adapted from Meyer et al. (1993)	Supervisory support had direct positive effects on nurses work engagement	Management of healthcare organizations should organize extensive training programmes for nurses and their supervisors to highlight the importance of nurturing workplace social support sources from supervisors and coworkers	STROBE: 20/22 JBI: 4b

Boamah [47] 2022 Canada	Cross sectional quantitative study	• 645 • Nurse faculty employed • 93.6% • 43.9	• Transformational leadership • Workplace culture • Burnout • Job satisfaction • COVID-19 impact	• Multifactor leadership questionnaire (MLQ-5X) from Bass and Avolio (2000) • Workplace culture scale designed specifically for the purposes of this study • Maslach burnout inventory-general survey • Global job satisfaction questionnaire by Hackman and Oldham (1976) • Specific scale created for the COVID-19 impact study	Transformational leadership had a strong and significant positive direct effect on workplace culture and job satisfaction and a negative effect on burnout	Nursing deans/directors should model transformational leadership in their daily behaviors, decisions and actions	STROBE: 20/22 JBI: 4b

Fish et al. [48] 2022 China and Australia	Comparative cross-sectional quantitative study	• 1837 • *n* = 730 (Australia) and *n* = 1107 (China) • Hospital nurses • 92.3% (A) and 97% (C) • 44.8 (A) and 32.5 (C)	• Burnout • Leadership support and adequate staffing • Patient safety	• The Maslach burnout inventory-human Service survey • Adapted subscales from practice environment scale of the nursing work index • Hospital survey on patient safety culture	Supportive leadership is crucial in both cultures to improve patient safety by mitigating aspects of burnout. However, staffing fit was particularly important in Australia, while in China, leadership more directly influences nurses' job satisfaction and well-being	Emotional exhaustion, depersonalization and personal accomplishment influenced patient safety distinctively across the countries. These findings inform interventions designed to reduce nurse burnout and improve patient safety internationally	STROBE: 20/22 JBI: 4b

Koç et al. [49] 2022 Turkey	Cross-sectional quantitative study	• 133 • Health care nurses • 92.2% • 32	• Toxic leadership • Emotional exhaustion • Intrinsic motivation	• Toxic leadership scale developed by Schmidt (2008) • Adaptation of the Maslach burnout inventory subscale • Intrinsic motivation inventory by Ryan (1982)	Toxic leadership is positively and significantly associated with emotional exhaustion	Intrinsic motivation plays a moderator role in the relationship between toxic leadership and emotional exhaustion. Managers exhibiting toxic leadership behaviors can cause severe problems both for organizations and employees	STROBE: 18.5/22 JBI: 4b

Parent-Lamarche et al. [50] 2022 Canada	Cross-sectional quantitative study	• 435 nurses, 92.93% and 27.19 • 116 nurse managers, 78% and 44.09	• Abusive leadership • Extra-role performance • Job emotional resources • Job emotional demands • Burnout • Autonomous motivation	• Tepper's abusive leadership scale (2000) • Job emotional resources adapted scale from Williams and Anderson (1991) • Job emotional demands adapted scale from the DISC 2.0 questionnaire by Van de Ven et al. (2008) • Maslach burnout inventory-general survey • Multidimensional work motivation scale by Gagné et al. (2015)	The significant interaction between abusive leadership and emotional resources indicates that the indirect relation of emotional resources on extra-role performance through autonomous motivation is moderated by abusive leadership. Abusive leadership significantly reduces nurses' autonomous motivation, which can lead to an increase in emotional exhaustion and cynicism (burnout indicators)	The results of this study may be particularly useful to promote effective leadership practices along with sufficient emotional resources in order to support nurses who have the autonomous motivation to go the extra mile	STROBE: 20/22 JBI: 4b

Srulovici and Janovich [51] 2022 Israel	Nested cross-sectional quantitative study	• 196 • Hospital nurses • 75% • 39.9	• Missed nursing care • Workload • Head nurse proactive leadership • Nurse exhaustion • Nurse vigor	• MISSCARE survey by Kalisch and Williams, (2009) • Work demand subscale by Haynes et al. (1999) • Proactivity Personality scale by Seibert et al. (1999) • Exhaustion subscale of the Maslach burnout inventory • Vigor subscale of the Utrecht work engagement scale	Moderate positive correlation was observed between proactive leadership levels of the head nurse and nurse' vigor level. Significant relationship between the proactive leadership of the head nurse and the level of exhaustion (burnout) of the nurses, specifically in the emotional exhaustion component	Emphasis should be placed on promoting and maximizing nurse motivation. This can be achieved by developing and implementing interventions of proactive leadership of head nurses	STROBE: 20/22 JBI: 4b

Westbrook et al. [52] 2022 USA	Cross-sectional quantitative study	• 248 • Inpatient and outpatient care facilities nurses • 83% • 42	• Servant leadership • Hindrance stressors • Burnout • Job satisfaction • Job performance • Turnover intentions	• Servant leadership survey by Van Dierendonck et al. (2017) • Adapted hindrance stressors scale by Cavanaugh et al. (2000) • Modified burnout scale from Babakus et al. (2009) • Bowling and Hammond's job satisfaction scale (2008) • Job performance scale by Babakus et al. (2009) • Turnover intentions scale by Babakus et al. (2009)	Servant leadership decreases nurse burnout while increasing job satisfaction	Servant leadership has a positive effect on the work environment and helps retain nursing professionals	STROBE: 18.5/22 JBI: 4b

Bartsch et al. [53] 2021 Germany	Cross-sectional quantitative study	• 470 • Healthcare assistants • 83.1%	• Key resources: Autonomy, interpersonal relationships, support, fair and authentic management, professional resources, supervisor support and transformational leadership • Key demands: workload, lack of formal rewards and work-life interference • Work engagement	• Copenhagen Psychosocial questionnaire • Utrecht work engagement scale	Neither fair management nor transformational leadership had a positive effect on work engagement during the pandemic. Positive associations were found between the key resources of autonomy, professional resources and interpersonal relationships and nurses' work engagement. Satisfaction with leaders significantly influences work engagement	The job demands-resources theory is suitable for explaining nurses' work engagement even in crisis times	STROBE: 20/22 JBI: 4b

Gasparino et al. [54] 2021 Brazil	Quantitative cross-sectional correlational study	• 1173 • Hospital nurses • 81.3% • 38.2	• Nursing practice environment • Safety climate and job satisfaction • Emotional exhaustion (burnout) • Intention to leave	• Practice environment scale • Safety attitudes questionnaire • Exhaustion subscale of the Maslach burnout inventory • Intention to leave questionnaire	The variables that most influenced the outcomes were nurse manager ability, leadership and support of nurses	Investment in the training of leaders, in the adequacy of resource and in physician–nurse relations will bring better results for patients, nursing professionals, and institutions	STROBE: 20/22 JBI: 4b

Ma et al. [55] 2021 Pakistan	Cross-sectional quantitative study	• 443 • Hospital nurses • 94% • 31	• Servant leadership • Psychological safety • Burnout	• Global servant leadership scale by Liden et al. (2015) • Psychological safety scale by Detert and Burris (2007) adapted from Edmondson (1999) • Maslach burnout inventory-general survey	Servant leadership and psychological safety have an inverse relationship with nurses' burnout	Servant leadership significantly reduces nurses' burnout, and psychological safety mediates this relationship	STROBE: 20/22 JBI: 4b

McKenna and Jeske [31] 2021 Ireland	Cross-sectional quantitative study	• 89 • Hospital nurses and nurse managers • 94.78% • 47.7	• Ethical leadership • Decision authority • Work engagement • Emotional exhaustion • Turnover intention	• Ethical leadership scale by Brown et al. (2005) • Decision authority subscale from Leiden quality of work life questionnaire by Maes et al. (1999) • Utrecht work engagement scale • Emotional exhaustion subscale from Maslach burnout inventory • Combination turnover intentions scale by Bozeman and Perrewé (2001) and Chalykoff and Kochan (1989)	Ethical leadership positively predicted decision authority and work engagement but not emotional exhaustion	The study found support for the positive role of ethical leadership in relation to decision authority and as a positive predictor of work engagement, negative predictor of emotional exhaustion and turnover intention among nurses	STROBE: 18.5/22 JBI: 4b

Dyrbye et al. [56] 2020 USA	Cross-sectional quantitative study	• 9683 • Healthcare nurses • 77.7% • 44	• Immediate supervisor leadership • Burnout • Organizational satisfaction	• Mayo Clinic leadership behavior score • Maslach burnout inventory adaptation • Organizational satisfaction measured with a single item	Supervisor scores in each dimension and composite leadership scores correlated with burnout and nurses' satisfaction. In logistic regression, each 1-point increase in leadership score was associated with a 7% decrease in odds of burnout and an 11% increase in odds of satisfaction of employees	Leadership qualities of immediate supervisors relate to burnout and satisfaction of nonphysician healthcare employees working in a large organization	STROBE: 19.5/22 JBI: 4b

Mostafa et al. [57] 2020 Romania	3-waves longitudinal prospective quantitative study	• 511/484/460 • Hospital nurses • 77.7% • 37.4	• Ethical leadership • Coworker undermining • Disengagement • Turnover intentions • Job satisfaction • Organizational commitment	• Ethical leadership scale by Brown et al.'s (2005) • Coworker undermining scale by Duffy et al.'s (2002) • Disconnection subscale Fron Oldenburg burnout inventory • Turnover intentions scale by O'Reilly et al. (1991) • Job satisfaction scale by Seashore et al. (1982) • Organizational commitments scale by Allen and Meyer (1990)	Ethical leadership was negatively correlated with disengagement, while coworker undermining was positively correlated with disengagement. Disengagement was positively correlated with turnover and negatively correlated with both job satisfaction and commitment	While ethical leaders can promote positive employee attitudes, their effectiveness is reduced in situations where coworker undermining exists	STROBE: 20/22 JBI: 3c

Waqas et al. [58] 2019 Pakistan	Cross-sectional quantitative study	• 300 • Hospital nurses • 91.5% • 28.10	• Transformational leadership • Personal concepts • Job challenges • Work engagement	• Transformational leadership questionnaire adapted from Leiter and Schaufeli (1996) • Personal concepts questionnaire based on Eagly (1967) • Job challenges questionnaire adapted from Ganster y Fusilier (1989) • Utrecht work engagement scale	Directly, transformational leadership did not demonstrate a significant relationship with work engagement. However, when mediating variables were introduced, this relationship became significant, indicating complete mediation	Transformational leaders reduced the stress of nurses which lead to self-confidence and caused them to perceive job demands as a challenge	STROBE: 19/22 JBI: 4b

Zaghini et al. [59] 2019 Italy	Cross-sectional quantitative multicentre study	• 479 • Hospital nurses • 74.5% • 41	• Patient's perception of nursing care quality • Nurses' satisfaction with leadership and management • Burnout • Interpersonal strain • Counterproductive work behaviors	• The caring behaviors scale by Piredda et al. (2017) • Nursing organizational health questionnaire • Maslach burnout inventory-general survey • Interpersonal strain at work scale • Nursing Counterproductive work behavior scale	The more satisfied nurses were with supervisors and management, the less they felt burnout. Satisfaction with leadership negatively influenced the emotional exhaustion of nurses. The perception of leadership also impacted on nurses' cynicism. Satisfaction with leadership was negatively associated with interpersonal strain	Effective leadership in the field of nursing can significantly reduce burnout and improve the quality of care perceived by patients, which underlines the importance of promoting leadership styles that support and strengthen work teams in the hospital environment	STROBE: 20/22 JBI: 4b

Chen and Chen [60] Taiwan 2018	Cross-sectional quantitative study	• 807 • Hospital nurses • 97.9% • 29.18	• Job demands • Job resources • Emotional intelligence • Leadership effectiveness • Burnout • Job satisfaction • Organizational commitment • Turnover intention	• Job demands adapted scale from Cavanaugh et al. (2000) • Job resources scale based on House scale (1981) • Mayer–Salovey–Caruso emotional intelligence test V.2.0 by Mayer et al. (2002) • Leadership effectiveness scale based on Hooijberg and Choi's scale (2001) • Emotional exhaustion scale adapted from Singh et al. (1994) • Job satisfaction scale adapted from Ashill and Rod's scale (2011) • Organizational commitment scale adapted from Mowday et al. (1979) • Turnover intention scale adapted from Mitchel (1981) and Good et al. (1996)	While stressors may aggravate nurses' burnout syndrome, supervisor support can have the opposite effect. The more nurses experience burnout conditions, the less job satisfaction and organizational commitment they perceive. Leadership effectiveness significantly enhances the buffering effect of job resources to burnout syndrome	The study highlights the significant role of leadership effectiveness and emotional intelligence in moderating the relationship between job demands, resources, and burnout. Effective leadership strategies can reduce burnout and improve retention among healthcare professionals	STROBE: 20/22 JBI: 4b

Enwereuzor et al. [61] 2016 Nigeria	Cross-sectional quantitative study	• 224 • Hospital nurses • 93.3% • 27.25	• Work engagement • Person–job fit • Transformational leadership	• Utrecht work engagement scale • Person–Job fit scale • Transformational leadership behavior inventory	Transformational leadership and person–job fit had a significant positive predictive relationship with work engagement. Simple slope analysis showed that person–job fit moderated the relationship between transformational leadership and work engagement such that transformational leadership was more positively related to work engagement for nurses with high person–job fit compared with those with low person–job fit	The more supervisors demonstrate transformational leadership style, the more followers are likely to be engaged in their work	STROBE: 18.5/22 JBI: 4b

Mauno et al. [62] 2016 Finland	Cross-sectional quantitative study	• 3466 • Health care nurses • 89% • 47.7	• Emotional labor • Work engagement • Compassion • Transformational leadership • Work ethic feasibility	• Emotional labor scale based on Zapf et al. Scale (1999) • Utrecht work engagement scale • Compassion subscale from public service motivation scale (PSM) by Kim et al. (2013) • Public service and global transformational leadership scale by de Carless et al. (2000) • Work ethic feasibility single-item measure developed by the authors	Higher transformational leadership was associated with higher work engagement	Transformational leadership has potential to improve engagement in nursing although it may not operate as a stress buffer	STROBE: 19.5/22 JBI: 4b

Fernet et al. [63] 2015 Canada	Cross-sectional quantitative study	• 637 • Public healthcare nurses • 88.4% • 29.63	• Transformational leadership • Job demands and resources • Work motivation • Psychological strain • Job attitudes • Job performance	• Global transformational leadership scale by Carless, Wearing y Mann (2000) • DISC 2.0 questionnaire by Van de Ven, Vlerick, and de Jonge (2008) • Multidimensional work motivation scale by Gagné et al. • Maslach burnout inventory-general survey • Occupational commitment questionnaire by Meyer et al. (1993) • Adapted scale from adaptación in-role performance subscale by Williams y Anderson (1991)	Transformational leadership is negatively related to job demands and psychological strain and positively related to job resources	Transformational leadership fosters optimal job functioning by enhancing perceived job resources, reducing job demands, and promoting autonomous motivation. This leadership style mitigates psychological strain, improves job attitudes, and boosts performance, underscoring its value in organizational settings	STROBE: 20/22 JBI: 4b

Laschinger et al. [64] 2015 Canada	Cross-sectional quantitative study	• 1009 • New graduate nurses • 92.5% • 27.43	• Authentic leadership • Areas of worklife • Occupational coping self-efficacy • Burnout • Mental health	• Authentic leadership questionnaire by Walumbwa et al. (2008) • Areas of worklife scale by Leiter y Maslach (2011) • Occupational coping self-efficacy scale by Pisanti et al. (2008) • Maslach burnout inventory-general survey • General health questionnaire by Goldberg y Williams (1988)	Authentic leadership had a positive effect on areas of worklife, which in turn had a positive effect on occupational coping self-efficacy, resulting in lower burnout, which was associated with poor mental health	Despite the significant mediation effect, the direct effect on health professionals' engagement persisted as statistically significant, indicating that the leadership factors are independently associated with employee engagement. Job resources were found to mediate the relationship between leadership factors and employee engagement	STROBE: 19.5/22 JBI: 4b

Hayati et al. [65] 2014 Iran	Cross-sectional quantitative study	• 240 • Hospital nurses • 77% • 25	• Transformational leadership • Work engagement	• Multifactor leadership questionnaire by Bass y Avolio (1997) • Utrecht work engagement scale	The highest mean and standard deviation values among all studied variables belong to transformational leadership and work engagement	Transformational leaders transfer their enthusiasm and high power to their subordinates. This manner can increase the power as a component of work engagement in workers	STROBE: 18/22 JBI: 4b

Bamford et al. [66] 2013 Canada	Cross-sectional quantitative study	• 280 • Hospital nurses • 93.5% • 43.4	• Authentic leadership • Areas of work life • Work engagement	• Authentic leadership questionnaire by Avolio et al. (2007) • Areas of worklife scale by Leiter y Maslach (2002) • Utrecht work engagement scale	Years of nursing experience and authentic leadership were significantly related to work engagement	Nurses who work for managers demonstrating higher levels of authentic leadership report a greater overall person–job match in the six areas of worklife and greater work engagement	STROBE: 19.5/22 JBI: 4b

Spence et al. [67] 2012 Canada	Cross-sectional quantitative study	• 342 • New graduate nurses • 92% • 28	• Authentic leadership • Bullying behaviors • Emotional exhaustion (burnout) • Job satisfaction • Turnover intentions	• Authentic leadership questionnaire by Avolio et al. (2007) • Negative acts questionnaire-revised by Einarsen and Hoel (2001) • Subscale from Maslach burnout inventory-general survey • Job satisfaction scale by Hackman and Oldham (1975) • Turnover intentions scale by Kelloway et al. (1999)	Authentic leadership was significantly correlated with all major study variables. Authentic leadership had a significant negative direct effect on workplace bullying experiences which in turn, had a significant positive effect on emotional exhaustion	Authentic leadership significantly reduces workplace bullying and burnout among newly graduated nurses, leading to higher job satisfaction and retention. Promoting authentic leadership behaviors is essential for fostering supportive work environments and mitigating turnover intentions	STROBE: 20/22 JBI: 4b

Salanova et al. [68] 2011 Portugal	Cross-sectional quantitative study	• 280 • Health care nurses • 79% • 34	• Transformational leadership • Self-efficacy • Vigor and dedication dimensions of work engagement • Extra-role performance	• Multifactor leadership questionnaire by Bass and Avolio (1990) • Self-efficacy scale specifically designed for this study • Utrecht work engagement scale • Organizational Citizenship Behavior scale by Morrison (1994)	Transformational leadership explained extra-role performance through self-efficacy and work engagement. A direct relationship between transformational leadership and work engagement was also found	Nurses' supervisors with a transformational leadership style enhance different “extra-role” performance in nurses and this increases hospital efficacy. They do so by establishing a sense of self-efficacy but also by amplifying their levels of engagement in the workplace	STROBE: 18.5/22 JBI: 4b

Bruyneel et al. [69] 2009 Belgium	Cross-sectional quantitative study	• 179 • Hospital nurses • 92.2% • 36.9	• Collaboration (joint practice) between nurses and physicians • Nurse manager ability, leadership and support of nurses • Staffing and resource adequacy • Burnout • Job satisfaction • Nurses' assessment of quality of care • Intention to leave the job in 1 year	• The original NWI-R Revised nursing work index by Aiken and Patrician (2000) • Maslach burnout inventory • Job satisfaction, nurses' perceptions of quality of care, and turnover intentions measured by 1 item	The factor staffing and resource adequacy was the main predictor of burnout. Nurse manager ability, leadership, and support of nurses were significant predictors of high job satisfaction	The study concludes that supportive and positive leadership significantly reduces burnout levels among nurses, especially in the aspect of emotional exhaustion	STROBE: 20/22 JBI: 4b

Argentero and Dell'Olivo [70] 2008 Italy	Cross-sectional quantitative study	• 180 • Intensive care nurses • 64% • 35	• Exhaustion, cynicism, professional efficacy • Areas of work life • Organizational changes • Management processes: leadership, skills development, work group cohesion	• Maslach burnout inventory-general survey • Areas of work life survey by Maslach y Leiter • Organizational changes perception scale • Organizational Check-Up survey	Burnout levels were strongly related to high demands, low control, low fairness, lack of social support, and individual disagreement on values in the workplace. High professional efficacy levels were instead correlated to professional reward and leadership involvement	Lack of social support from coworkers (group cohesion) and from one's leadership can be associated with both emotional exhaustion and cynicism	STROBE: 20/22 JBI: 4b

Greco et al. [71] 2006 Canada 38	Cross-sectional quantitative study	• 322 • Hospital nurses • 97.2% • 42	• Leader empowering behaviors • Structural empowerment • Areas of worklife • Work engagement • Burnout	• The leader empowering behavior scale by Hui (1994) • Conditions of work effectiveness Questionnaire-II by Laschinger et al. (2001) • Areas of worklife scale by Leiter y Maslach (2004) • Maslach burnout inventory-general survey	Leader empowering behavior had an indirect effect on emotional exhaustion through structural empowerment and overall fit in the six areas of work life	Leader's empowering behaviors can enhance person–job fit and prevent burnout. The results reinforce the importance of leadership in creating engaging, satisfying workplaces for nurses	STROBE: 19/22 JBI: 4b

**Table 2:** Methodological quality of the studies; items of STROBE scale [72].

	Year	1	2	3	4	5	6	7	8	9	10	11	12	13	14	15	16	17	18	19	20	21	22	Total
Ding and Cao [35]	2024	✓	✓	✓	✓	✓	✓	✓	✓	∼	✓	✓	✓	✓	✓	✓	✓	✓	✓	✓	✓	✓	✓	21.5
Kohnen et al. [36]	2024	✓	✓	✓	∼	✓	✓	✓	✓	∼	✓	✓	✓	✓	✓	✓	✓	✗	✓	✓	✓	✓	✓	20
Uluturk et al. [37]	2024	✓	✓	✓	∼	✓	✓	✓	✓	∼	✓	✓	✓	✓	✓	✓	✓	✗	✓	✓	✓	✗	✓	19
Ahmed et al. [38]	2023	✓	✓	✓	∼	✓	✓	✓	✓	∼	✓	✓	✓	✓	✓	✓	✓	✓	✓	✓	✓	✓	✓	21
Apaydin et al. [39]	2023	✓	✓	✓	∼	✓	✓	✓	✓	∼	✓	✓	✓	✓	✓	∼	✓	✗	✓	✓	✓	✓	✓	19.5
Aung Po et al. [40]	2023	✓	✓	✓	∼	✓	✓	✓	✓	∼	✓	✓	✓	✓	✓	∼	✓	✗	✓	✓	✓	✓	✓	19.5
Buttigieg et al. [41]	2023	✓	✓	✓	∼	✓	✓	✓	✓	∼	✓	✓	✓	✓	✓	✓	✓	✓	✓	✓	✓	✓	✓	21
Forster and Koob [42]	2023	✓	✓	✓	∼	✓	✓	✓	✓	∼	✓	✓	✓	✓	✓	✓	✓	✗	✓	✓	✓	✓	✓	20
Giallouros et al. [43]	2023	✓	✓	✓	∼	✓	✓	✓	✓	∼	✓	✓	✓	✓	✓	∼	✓	✗	✓	✓	✓	✓	✓	19.5
Hasan et al. [44]	2023	✓	✓	✓	∼	✓	✓	✓	✓	∼	✓	✓	✓	✓	✓	∼	✓	✗	✓	✓	✓	✓	✓	19.5
Palvimo et al. [45]	2023	✓	✓	✓	∼	✓	✓	✓	✓	∼	✓	✓	✓	✓	✓	∼	✓	✗	✓	✓	✓	✓	✓	19.5
Uddin [46]	2023	✓	✓	✓	∼	✓	✓	✓	✓	∼	✓	✓	✓	✓	✓	✓	✓	✗	✓	✓	✓	✓	✓	20
Boamah [47]	2022	✓	✓	✓	∼	✓	✓	✓	✓	∼	✓	✓	✓	✓	✓	✓	✓	✗	✓	✓	✓	✓	✓	20
Fish et al. [48]	2022	✓	✓	✓	∼	✓	✓	✓	✓	∼	✓	✓	✓	✓	✓	✓	✓	✗	✓	✓	✓	✓	✓	20
Koç et al. [49]	2022	✓	✓	✓	∼	✓	✓	✓	✓	∼	✓	✓	✓	✓	✓	∼	✓	✗	✓	✓	✓	✗	✓	18.5
Parent-Lamarche et al. [50]	2022	✓	✓	✓	∼	✓	✓	✓	✓	∼	✓	✓	✓	✓	✓	✓	✓	✗	✓	✓	✓	✓	✓	20
Srulovici and Janovich [51]	2022	✓	✓	✓	∼	✓	✓	✓	✓	∼	✓	✓	✓	✓	✓	✓	✓	✗	✓	✓	✓	✓	✓	20
Westbrook et al. [52]	2022	✓	✓	✓	∼	✓	✓	✓	✓	∼	✓	✓	✓	✓	✓	∼	✓	✗	✓	✓	✓	✗	✓	18.5
Bartsch et al. [53]	2021	✓	✓	✓	∼	✓	✓	✓	✓	∼	✓	✓	✓	✓	✓	✓	✓	✗	✓	✓	✓	✓	✓	20
Gasparino et al. [54]	2021	✓	✓	✓	∼	✓	✓	✓	✓	∼	✓	✓	✓	✓	✓	✓	✓	✗	✓	✓	✓	✓	✓	20
Ma et al. [55]	2021	✓	✓	✓	∼	✓	✓	✓	✓	∼	✓	✓	✓	✓	✓	✓	✓	✗	✓	✓	✓	✓	✓	20
McKenna and Jeske [31]	2021	✓	✓	✓	∼	✓	✓	✓	✓	∼	✓	✓	✓	✓	✓	∼	✓	✗	✓	✓	✓	✗	✓	18.5
Dyrbye et al. [56]	2020	✓	✓	✓	∼	✓	✓	✓	✓	∼	✓	✓	✓	✓	✓	∼	✓	✗	✓	✓	✓	✓	✓	19.5
Mostafa et al. [57]	2020	✓	✓	✓	✓	✓	✓	✓	✓	∼	✓	✓	✓	✓	✓	∼	✓	✗	✓	✓	✓	✗	✓	20
Waqas et al. [58]	2019	✓	✓	✓	∼	✓	∼	✓	✓	∼	✓	✓	✓	✓	✓	∼	✓	✗	✓	✓	✓	✓	✓	19
Zaghini et al. [59]	2019	✓	✓	✓	∼	✓	✓	✓	✓	∼	✓	✓	✓	✓	✓	✓	✓	✗	✓	✓	✓	✓	✓	20
Chen and Chen [60]	2018	✓	✓	✓	∼	✓	✓	✓	✓	∼	✓	✓	✓	✓	✓	✓	✓	✗	✓	✓	✓	✓	✓	20
Enwereuzor et al. [61]	2016	✓	✓	✓	∼	✓	✓	✓	✓	∼	✓	✓	✓	✓	✓	∼	✓	✗	✓	✓	✓	✗	✓	18.5
Mauno et al. [62]	2016	✓	✓	✓	∼	✓	✓	✓	✓	∼	✓	✓	✓	✓	✓	∼	✓	✗	✓	✓	✓	✓	✓	19.5
Fernet et al. [63]	2015	✓	✓	✓	∼	✓	✓	✓	✓	∼	✓	✓	✓	✓	✓	✓	✓	✗	✓	✓	✓	✓	✓	20
Laschinger et al. [64]	2015	✓	✓	✓	∼	✓	✓	✓	✓	∼	✓	✓	✓	✓	✓	∼	✓	✗	✓	✓	✓	✓	✓	19.5
Hayati et al. [65]	2014	✓	✓	✓	∼	✓	✓	✓	✓	∼	✓	✓	∼	✓	✓	∼	✓	✗	✓	✓	✓	✗	✓	18
Bamford et al. [66]	2013	✓	✓	✓	∼	✓	✓	✓	✓	∼	✓	✓	✓	✓	✓	∼	✓	✗	✓	✓	✓	✓	✓	19.5
Spence et al. [67]	2012	✓	✓	✓	∼	✓	✓	✓	✓	∼	✓	✓	✓	✓	✓	✓	✓	✗	✓	✓	✓	✓	✓	20
Salanova et al. [68]	2011	✓	✓	✓	∼	✓	✓	✓	✓	∼	✓	✓	✓	✓	✓	∼	✓	✗	✓	✓	✓	✗	✓	18.5
Bruyneel et al. [69]	2009	✓	✓	✓	∼	✓	✓	✓	✓	∼	✓	✓	✓	✓	✓	✓	✓	✗	✓	✓	✓	✓	✓	20
Argentero and Dell'Olivo [70]	2008	✓	✓	✓	∼	✓	✓	✓	✓	∼	✓	✓	✓	✓	✓	✓	✓	✗	✓	✓	✓	✓	✓	20
Greco et al. [71]	2006	✓	✓	✓	∼	✓	✓	✓	✓	✓	✓	✓	✓	✓	✓	∼	✓	✗	✓	✓	✓	✗	✓	20

*Note:* ✓: study fully meets the item; ✗: study does not meet the item; ∼: study partially meets the item.

## Data Availability

Data sharing is not applicable to this article as no datasets were generated or analyzed during the current study.
